# Leisure time physical activity in middle age predicts the metabolic syndrome in old age: results of a 28-year follow-up of men in the Oslo study

**DOI:** 10.1186/1471-2458-7-154

**Published:** 2007-07-12

**Authors:** Ingar Holme, Serena Tonstad, Anne Johanne Sogaard, Per G Lund Larsen, Lise Lund Haheim

**Affiliations:** 1 Centre of Preventive Medicine, Ullevål University Hospital, 0407 Oslo, Norway; 2 Depart.of Epidemiology, Norwegian Institute of Public Health, 0403 Oslo, Norway; 3 Depart.of Epidemiology, Institute of General Practice and Community Medicine.University of Oslo, 0318 Oslo, Norway; 4 Norwegian Knowledge Centre for the Health Services, 0130 Oslo, Norway

## Abstract

**Background:**

Data are scarce on the long term relationship between leisure time physical activity, smoking and development of metabolic syndrome and diabetes. We wanted to investigate the relationship between leisure time physical activity and smoking measured in middle age and the occurrence of the metabolic syndrome and diabetes in men that participated in two cardiovascular screenings of the Oslo Study 28 years apart.

**Methods:**

Men residing in Oslo and born in 1923–32 (n = 16 209) were screened for cardiovascular diseases and risk factors in 1972/3. Of the original cohort, those who also lived in same area in 2000 were invited to a repeat screening examination, attended by 6 410 men. The metabolic syndrome was defined according to a modification of the National Cholesterol Education Program criteria. Leisure time physical activity, smoking, educational attendance and the presence of diabetes were self-reported.

**Results:**

Leisure time physical activity decreased between the first and second screening and tracked only moderately between the two time points (Spearman's ρ = 0.25). Leisure time physical activity adjusted for age and educational attendance was a significant predictor of both the metabolic syndrome and diabetes in 2000 (odds ratio for moderately vigorous versus sedentary/light activity was 0.65 [95% CI, 0.54–0.80] for the metabolic syndrome and 0.68 [0.52–0.91] for diabetes) (test for trend P < 0.05). However, when adjusted for more factors measured in 1972/3 including glucose, triglycerides, body mass index, treated hypertension and systolic blood pressure these associations were markedly attenuated. Smoking was associated with the metabolic syndrome but not with diabetes in 2000.

**Conclusion:**

Physical activity during leisure recorded in middle age prior to the current waves of obesity and diabetes had an independent predictive association with the presence of the metabolic syndrome but not significantly so with diabetes 28 years later in life, when the subjects were elderly.

## Background

The prevalence of diabetes mellitus and its precursor, the metabolic syndrome has increased markedly in many countries [1], including Norway, and diabetes is becoming common worldwide [2,3]. Obesity and its duration are major risk factors for the development of type 2 diabetes. A general trend towards a more sedentary society with the advent of modern electronic equipment and computers, more time spent in sedentary activities as television viewing as well as reduced physical activity at work and at leisure may be major causative components in this development [4-8]. A change in dietary habits has also taken place in the same time period [9]. Cigarette smoking, which is associated with a decreased body mass index (BMI) in most populations [10] but increased central obesity [11], has steadily declined in Western societies and smoking cessation has contributed to some of the weight gain that has been seen in certain populations [12,13]. Thus, except for smoking, several of the major lifestyle habits have developed unfavourably with regard to the incidence of obesity, the metabolic syndrome and diabetes. Within populations, however, large differences have been observed depending on the education and socioeconomic status of individuals [14].

A number of studies have reported on the role played by these key risk factors in regard to the development of the metabolic syndrome and diabetes [4-7,15-19]. We were interested in exploring prospectively whether lifestyle habits established nearly three decades previously influenced the subsequent occurrence of diabetes and the metabolic syndrome in an elderly cohort. The re-examination in the year 2000 of men screened for cardiovascular risk factors and diseases in the Oslo Study in 1972/3 [20] provided the opportunity to examine the relationship between leisure time physical activity (LTPA) and smoking in 1972/3 and the metabolic syndrome and diabetes 28 years later.

## Methods

The design and methods of the Oslo Study of 1972/3 have been described earlier [20]. In brief, all men born in 1923–32 and residing in the city of Oslo were invited to a screening examination for cardiovascular diseases and risk factors. A total of 16 209 men aged 40–49 years at baseline attended, which represented 63% of this age group. Height, weight, and blood pressure were measured and a blood sample taken in the non-fasting state was used for measurements of total serum cholesterol, triglycerides and glucose. Time since the last meal was recorded. Participants filled in a questionnaire regarding prevalent diseases and symptoms of cardiovascular origin and diabetes, smoking habits, degree of physical activity at work and at leisure and a few questions about mental stress. LTPA was defined in four groups as follows: sedentary/light: usually reading, watching television or other sedentary occupations at leisure; moderate: walking, bicycling or other forms of physical activity including walking or bicycling to and from the place of work and a Sunday walk totalling at least four hours a week; moderately vigorous: exercise, sports, heavy gardening and similar activities totalling at least 4 hours a week; vigorous: hard training or competition sports regularly several times a week. The respondent was asked to use the average amount of activity, if activity varied much for example between summer and winter. This question has been found to show solid correlation to physical fitness and have good predictive validity in prospective studies [21-23].

In 2000–2001 the conductance of the Oslo Health Study 2000–2001 (abbreviated as HUBRO), a population-based survey of selected birth cohorts living in Oslo in 2000–2001 [24] took place. Additional to that study all men originally invited to the Oslo Study in 1972/3 and resident in Oslo or the neighbouring county of Akershus were invited to a repeat screening examination (designated Oslo II)[25]. Height, weight, and blood pressure were measured again and non-fasting blood samples were taken for measurements of serum total cholesterol, triglycerides and glucose. This time high density cholesterol (HDL) cholesterol was also included. Time since the last meal was recorded. Attendees filled in two questionnaires covering smoking habits and the same questions concerning LTPA as they did in 1972/3 as well as a number of other issues including educational attendance in number of years.

Some of the participants who met the criteria for inclusion in Oslo II were invited to the screening through HUBRO while others were invited through another study promoting physical activity in the community [26]. Both studies used the same screening procedures and questionnaires as Oslo II. In total 1 095 men from these studies participated and their data were later included in the Oslo II study database. Moreover 813 eligible subjects for Oslo II were participating in three ongoing clinical trials. These men were invited to fill in the same questionnaires that were used in Oslo II at the close of the trials and they provided fasting blood sample values which accordingly were not adjusted to eight hours since last meal. The Norwegian Data Inspectorate allowed the data and the measurements in these five studies to be added to the Oslo II database.

Men who were dead or had emigrated (n = 1 655), men living outside the catchment area of Oslo and Akershus (n = 1 278) and men with unknown addresses (n = 2 944) were excluded from the Oslo II screening leaving 10 328 candidates for the study. Of these 6 410 of the men who attended the baseline study, also attended the follow up, resulting in an attendance proportion of 62.0%. Finally 6 382 men without reported diabetes in 1972/3 (n = 28 had diabetes or non fasting glucose ≥ 11.1 mmol/l) and with systolic blood pressure measurements both in 1972/3 and in 2000 constituted the population for analysis.

We based the definition of the presence or absence of the metabolic syndrome in year 2000 based on a modification of the National Cholesterol Education Program Adult Treatment Program III criteria (NCEP III) [27]. Because of the unavailability of waist circumference, BMI was used to replace it with a cut-off of a BMI ≥30.0 kg/m^2 ^corresponding to about a waist circumference of 102 cm. Furthermore fasting and non-fasting glucose levels were adjusted to correspond to eight hours since last meal as the criteria require fasting levels. Triglyceride levels were also adjusted to eight hours since the last meal. In summary the five criteria of the metabolic syndrome were defined as follows: triglycerides ≥1.7 mmol/l adjusted for the last meal, glucose ≥6.1 mmol/l adjusted for the last meal, BMI ≥30.0 kg/m^2^, blood pressure ≥135/85 mmHg (use of antihypertensive medication was not included in the hypertension definition), and HDL cholesterol <1.03 mmol/l. The metabolic syndrome was defined as the presence of at least three out of five criteria. We could not define MS in 1972/3 according to NCEP III criteria because we lacked measurements of HDL-C.

Smoking was categorized as never (reference), previous and current. LTPA was grouped as the same four groups at both screenings. In year 2000 the definition of diabetes included self reported diabetes, men who took oral antidiabetic agents, used insulin or had a non fasting glucose ≥ 11.1 mmol/l.

### Ethics and approvals

All the participants of the Oslo Study have given their written signed consent. The Norwegian Data Inspectorate has approved the Oslo Study, it has been cleared by the Regional Committee for Medical Research Ethics and it has been conducted in full accordance with the World Medical Association Declaration of Helsinki.

### Statistics

Because blood samples were non-fasting (except for subjects that participated in one of the three randomised trials mentioned above), second order regression equations were fitted between level of triglycerides and of glucose and time since last meal. The values were adjusted to eight hours after the last meal. Odds ratios (OR) with 95% confidence limits were calculated by logistic regression analyses. The metabolic syndrome in 2000 and diabetes in 2000 were dependent variables. Age, length of education in years, smoking, LTPA, levels of glucose and triglycerides, body mass index and systolic blood pressure were independent variables. Likelihood ratio tests were used to test the significance of the factors. Test of trend for LTPAwas done assuming interval scale of the variable. In 2000 the question regarding LTPA was answered by only 66% of attendees since HUBRO did not include this question in its survey. Thus the comparison of LTPA in 2000 to 1972/3 had correspondingly fewer data points.

Spearman's correlation coefficient was used to estimate the relationship of LTPA in 2000 to LTPA in 1972/3 while Pearsons' correlation coefficient was used for the relationship of BMI in 2000 to BMI in 1972/3. A Chi square test was used to test for changes in smoking habits. The SPSS 13.0 software program was used for all analyses.

## Results

### Changes in exposure factors

Cigarette smoking showed a significant change between the initial and subsequent screenings with a reduction from a prevalence of 43.9% to 17.6% (P < 0.0001; table 1). In contrast rather small changes in the distribution of LTPA occurred. Vigorous or moderately vigorous activity was reported by 22.8% in 1972/3 compared to 18.5% in 2000 (P = 0.015). The degree of tracking between the two points measured by the correlation coefficient was only slight in regard to LTPA (Spearman's ρ = 0.25), whereas the correlation coefficient for BMI at the two time points was much higher (Pearson's r = 0.69).

**Table 1:** Characteristics of men that attended the screening examinations in 1972/3 and 2000 (n = 6 382)

**Risk factor**		**n**	**%**	**n**	**%**
		Year 1972/3		Year 2000	
Smoking	Never	1 602	25.1	1 595	26.7
	Previous	1 979	31	3 330	55.7
	Current	2 801	43.9	1 050	17.6
	Missing	-	-	407	-
Leisure time physical activity	Sedentary/light	1 111	17.4	754	17.9
	Moderate	3 810	59.7	2 700	63.7
	Moderately vigorous	1 317	20.6	735	17.3
	Vigorous	141	2.2	50	1.2
	Missing	3	-	2 143	-
Metabolic syndrome*		NA	NA	1 597	25
Antihypertensive drugs		-	-	2018	33.8
Diabetes§/P >		-	-	584	9.2
Blood pressure **≥ 130/85 mmHg**		2 568	40.2	2 777	43.5
Blood glucose **≥ 6.1 mmol/l**		1 497	23.5	1 073	19.4
BMI **≥ 30.0 kg/m**^2^		162	2.5	797	12.5
Triglycerides **≥ 1.7 mmol/l**		2 416	37.9	2 231	40.4
HDL cholesterol **< 1.03 mmol/l**		NA	NA	910	14.3

*The metabolic syndrome was defined if at least 3 out of 5 of the criteria were present: triglycerides ≥ 1.7 mmol/l, glucose ≥ 6.1 mmol/l, BMI ≥ 30.0 kg/m^2^, blood pressure ≥ 130/85 mmHg, HDL cholesterol < 1.03 mmol/l.

§Diabetes was defined as self reported diabetes, use of antidiabetic medication including insulin or non-fasting glucose ≥ 11.1 mmol/l.

NA: inability to define MS according to NCEP III criteria due to missing HDL-C

### Univariate relationships between exposure factors and outcome

There was a clear relationship between smoking in 1972/3 and the metabolic syndrome in 2000 (table 2). LTPA in 1972/3 was also strongly related to the prevalence of the metabolic syndrome in 2000 demonstrating a steep negative dose-response relationship, although precision was low at the highest level of physical activity (table 2).

**Table 2:** Odds ratios of the metabolic syndrome and diabetes in 2000 according to smoking (separate model) and leisure time physical activity (separate model) in 1972/3 adjusted for age and years of education

	Metabolic syndrome*	**Diabetes**				
**Risk factor in 1972/3**	**Cases**	**%**	**Odds ratio (95% CL)**	**Cases**	**%**	**Odds ratio (95% CL)**

**Smoking**						
Never (reference)	354	22.1		135	9.2	
Previous	476	24.1	1.12 (0.95–1.32)	187	10.3	1.12 (0.88–1.42)
Current	767	27.4	1.29 (1.11–1.51)	262	11.1	1.16 (0.92–1.45)
**Leisure time physical activity**						
Sedentary/light	337	30.3		127	13.4	
Moderate	967	25.4	0.83 (0.71–0.98)	344	10.1	0.75 (0.60–0.94)
Moderately vigorous	269	20.4	0.65 (0.54–0.80)	108	9.1	0.68 (0.52–0.91)
Vigorous	24	17.0	0.46 (0.28–0.74)	5	4.0	0.28 (0.11–0.71)

* The metabolic syndrome in year 2000 was defined if at least 3 out of 5 of the criteria were present: triglycerides ≥ 1.7 mmol/l, glucose ≥ 6.1 mmol/l, BMI ≥ 30.0 kg/m^2^, blood pressure ≥ 130/85 mmHg, HDL cholesterol < 1.03 mmol/l.

The prevalence of diabetes was 9.2% in 2000. There was no detectable relationship between smoking in 1972/3 and diabetes in 2000. LTPA in 1972/3 was a powerful predictor of diabetes in 2000 (table 2).

### Multivariate analyses

Table 3 shows the association of smoking and LTPA in 1972/3 with the metabolic syndrome in 2000 when age, length of education, smoking, LTPA, levels of glucose and triglycerides, BMI, treated hypertension and systolic blood pressure were included in the model. LTPA in 1972/3 predicted the metabolic syndrome in 2000 but clearly weaker than without adjustments for smoking, glucose, body mass index, triglycerides, treated hypertension and systolic blood pressure. Table 3 further shows that there was a significant association between smoking and the metabolic syndrome but not with diabetes in 2000. LTPA in 1972/3 was no longer significantly associated with diabetes, mainly due to the attenuating effects of glucose levels and BMI in 1972/3 (data not shown).

**Table 3:** Multiple logistic regression analysis of the metabolic syndrome and diabetes in year 2000 in relation to smoking, leisure time physical activity (both included as cofactors) adjusted for age, years of education, glucose, triglycerides, body mass index, treated hypertension and systolic blood pressure in 1972/3

		Metabolic syndrome*		**Diabetes**	
**Risk factor in 1972/3**		**Odds ratio (95% CL)**	**P-value§**	**Odds ratio (95% CL)**	**P-value§**

**Smoking**			0.001		NS
	Never (reference)				
	Previous	1.01 (0.85–1.21)		1.02 (0.79–1.31)	
	Current	1.30 (1.10–1.53)		1.17 (0.92–1.48)	
**Leisure time physical activity**					
	Sedentary/light		0.039		0.129
	Moderate	0.98 (0.83–1.17)		0.87 (0.68–1.10)	
	Moderately vigorous	0.83 (0.67–1.02)		0.87 (0.64–1.17)	
	Vigorous	0.73 (0.44–1.22)		0.42 (0.17–1.06)	

*The metabolic syndrome in year 2000 was defined if at least 3 out of 5 of the criteria were present: triglycerides ≥ 1.7 mmol/l, glucose ≥ 6.1 mmol/l, BMI ≥ 30.0 kg/m^2^, blood pressure ≥ 130/85 mmHg, HDL cholesterol <1.03 mmol/l. § Test of trend

## Discussion

This study showed that LTPA reported by men who were middle-aged in 1972/3 was a significant predictor of diabetes in 2000 when the men were elderly. There was also a consistent relationship between LTPA and the metabolic syndrome, whereas the degree of tracking was rather low. These findings primarily confirm previous reports regarding physical activity and diabetes [4-7] but do so for the first time regarding physical activity and the metabolic syndrome in a cohort of men followed from middle to old age for a span of 28 years. Furthermore our finding of a clear relationship between LTPA and the metabolic syndrome in elderly men after a redistribution of the subjects in the different categories of activity reinforces the notion of causality in the relationship between LTPA and the metabolic syndrome. Even after adjustment for several of the criteria for the metabolic syndrome in 1972/3, LTPA in 1972/3 remained significantly associated with the metabolic syndrome in 2000. Thus it seems that an increased activity pattern at leisure time reported during middle age predicts a reduced proportion of subjects with the metabolic syndrome nearly three decades later.

The prevalence of current smoking was markedly reduced between the initial and second examinations. This is probably partly due to the premature mortality of smokers. In addition, falling rates of smoking have been a general trend in many Western countries. Body weight increases after smoking cessation and thus the prevalence of the metabolic syndrome may be higher in quitters than in never smokers or in current smokers [13]. Persistent smokers usually have a lower BMI than never smokers [10] and could be expected to have a lower prevalence of the metabolic syndrome if they do not quit. However, current smoking in 1972/3 was associated with the prevalence of the metabolic syndrome in 2000 since many quit smoking during these years. Smoking is an established correlate and probably a cause of increased insulin resistance and diabetes [17,18,28].

LTPA in 1972/3 was a strong predictor for diabetes as well as for the metabolic syndrome in 2000. However, these relationships were greatly attenuated after adjustment for levels of glucose and triglycerides, BMI, treated hypertension and systolic blood pressure, all of which were predictors of diabetes and the metabolic syndrome (data not shown). The relationships were graded, reinforcing the causality of LTPA as risk factor for development of the metabolic syndrome and diabetes. Since the metabolic syndrome is a significant predictor of cardiovascular events in older individuals [29], the relationships of LTPA in particular indicate that the metabolic syndrome is preventable and this may contribute to lowering of risk of cardiovascular disease if effectively treated. Several lines of evidence indicate that insulin resistance is the primary cause of the metabolic syndrome. One mechanism by which physical activity lowers the risk of the metabolic syndrome is that participation in even non vigorous activity is associated with higher insulin sensitivity [30].

### Strengths and limitations

These findings confirm the results of a number of observational studies with shorter follow up on the relation of physical activity to the metabolic syndrome [31,32]. A major strength of this study is its long term follow up with baseline measurements that were obtained in relatively lean individuals prior to the current wave of obesity in the Western world. The statistical power of the study is reasonably high because it included more than 500 cases with diabetes or the metabolic syndrome in 2000. It is highly probable that a selective survival pattern took place during the long follow-up, so that men that rapidly transferred into a diabetic condition died first. It is therefore likely that the relationship of LTPA to diabetes has been conservatively estimated.

There are several methodological limitations. The data were collected from several studies, however, a subgroup analysis including only the 4 490 men in the core Oslo II study revealed similar trends for the relationships between LTPA and the metabolic syndrome and diabetes as for the total dataset (data not shown).

The study has a repeated cross sectional design with 28 years between the two screenings. Diseases that started prior to 2000 may have affected biological and anthropometric indices as well as lifestyle factors.

Because we only had non-fasting laboratory tests, we estimated the triglyceride and glucose levels by using regression equations that adjusted to 8 hours since the last meal. This adjustment is only approximately valid as a mean for the group, so caution is needed in comparison with other studies using fasting values. Because of this adjustment levels of triglycerides and glucose were adjusted downwards by about 0.2–0.3 mmol/l, which is usually less than the difference in measurements taken in the fasting or in the non-fasting state. Thus, we may underestimate somewhat the prevalence of metabolic syndrome. Length of fasting was not significantly associated to LTPA levels at baseline (P = 0.09). We did not exclude subjects with the metabolic syndrome in 1972/3 when we analyzed the relation between LTPA to the metabolic syndrome in 2000. A reanalysis of the OR for the metabolic syndrome in 2000 (Table 3) when excluding these subjects gave almost identical results (data not shown).

Our definition of diabetes differed in 1972/3 and in 2000, since we did not ask about the use of antidiabetic medication in 1972/3. When we redefined diabetes in 2000 by including only self reported cases and those with non-fasting glucose ≥ 11.1 mmol/l, we found similar non-significant results as in Table 3, with an OR of 1.01 (95% CI 0.95, 0.68; P for trend = 0.26) in the three LTPA groups.

A certain selection in regard to tracking of LTPA took place as those who answered the question on LTPA at both screenings smoked less and had a longer education than other subjects. The LTPA questions may have been interpreted differently by the men in 1972/3 in their middle-age compared to in 2000, however, this does not affect the predictivity of the questions in regard to the metabolic syndrome. We do not have data on dietary habits and alcohol intake in 1972/3, both factors which have been shown to influence the metabolic syndrome in previous studies [16].

Subjects that attended in 2000 tended to have lower body weight, height, systolic and diastolic blood pressure, total cholesterol, triglycerides and glucose values in 1972/3 than non-attendees as well as a lower prevalence of smoking (data not shown). As we do not have mortality data we cannot estimate how much premature mortality contributed to these differences.

## Conclusion

We found that low LTPA in 1972/3 predicted the metabolic syndrome and diabetes in 2000, both directly and independently after adjustment for age and length of education. However, the relationships were attenuated when adjusted for factors such as glucose and BMI. Smoking in 1972/3 predicted the metabolic syndrome but not diabetes in year 2000. Our study supports the notion that the metabolic syndrome is preventable in older men.

## Competing interests

The author(s) declare that they have no competing interestss.

## Authors' contributions

IH was responsible for statistical analysis and had the leading role in writing up the manuscript. ST made a major contribution to the discussion and writing of the manuscript. AJS participated in the design stage of the study and contributed to a major degree in commenting and writing of the manuscript. PGLL made a major contribution to the design and data management of the study. LLH conceived the Oslo II study, was the study coordinator and contributed to the manuscript. All authors approved the manuscript.

## Pre-publication history

The pre-publication history for this paper can be accessed here:


